# Case Report: Diabetic nephropathy aggravates the progression and prognosis of COVID-19-associated acute limb ischemia

**DOI:** 10.12688/f1000research.54193.1

**Published:** 2021-07-16

**Authors:** Decsa Medika Hertanto, Henry Sutanto, Soebagijo Adi

**Affiliations:** 1 Department of Internal Medicine, Airlangga University, Surabaya, Jawa Timur, 60132, Indonesia; 2 Department of Internal Medicine, Dr. Soetomo Hospital, Surabaya, Jawa Timur, 60286, Indonesia; 3 Department of Cardiology, Maastricht University, Maastricht, Limburg, 6229ER, The Netherlands; 4 Department of Physiology and Biophysics, SUNY Downstate Medical Center, Brooklyn, New York, 11203, USA; 5 Division of Endocrinology, Diabetes and Metabolism, Department of Internal Medicine, Dr. Soetomo Hospital, Surabaya, Jawa Timur, 60286, Indonesia

**Keywords:** acute limb ischemia; COVID-19; diabetes mellitus; diabetic nephropathy; limb amputation; hypercoagulability; vascular thrombosis, case report

## Abstract

Hypercoagulation is a hallmark of both the novel coronavirus disease (COVID-19) and type 2 diabetes mellitus (T2DM). It increases the risk for vascular thrombosis, including peripheral artery diseases. Among others, acute limb ischemia (ALI) is one of most common complications that requires immediate and prompt treatments to reduce morbidity and mortality. However, the complex interplay between COVID-19, T2DM and its complications (e.g., diabetic nephropathy), and ALI creates a great challenge in the management of the disease. Here, we present a case of a 59-year-old diabetic female with progressive pain in her left leg in the last five years, which was significantly intensified following COVID-19 diagnosis. Bluish coloration, numbness and functional impairments were observed during examinations with no palpable pulsation on left posterior tibial and dorsalis pedis arteries. The patient also had diabetic nephropathy (stage III), hypoalbuminemia, anemia and a urinary tract infection that complicated the management of the disease. Due to the excruciating pain and the worsening of the limb conditions, right leg revascularization and left leg amputation were performed at day 14 after admission. Following the surgeries, no more pain was observed and patient was discharged for further follow-up at the outpatient clinic.

## Introduction

The 2019 coronavirus disease (COVID-19) is an infectious disease caused by the severe acute respiratory syndrome-associated coronavirus type 2 (SARS-CoV-2). It was first encountered in Wuhan city, Hubei province, China in late 2019 and since early 2020, it has been declared a pandemic by the World Health Organization (CDC, 2021; WHO, 2021). The viral disease infects healthy people through respiratory or micro droplets released by infected individuals during sneezing, speaking or coughing (WHO, 2021). Following its entry into the respiratory tract of the contracted individuals, the virus binds to the angiotensin converting enzyme type 2 (ACE-2) receptors in the alveolar cells type 2 to interact with the host’s cellular immunity ( Hertanto, Sutanto, & Wungu, 2021). In the first months of the pandemic, the virus was believed to solely affect the respiratory system, causing severe pneumonia. However, it is now understood that SARS-CoV-2 affects various organs and subsequently worsens other comorbidities, including type 2 diabetes mellitus (T2DM) ( Mokhtari *et al*., 2020). Interestingly, T2DM is also one of the biggest contributors to the worsening of COVID-19 severity, creating a vicious cycle. The COVID-19-associated mortality has been reported to be doubled in patients with T2DM as compared to those without ( Aggarwal *et al*., 2020; Parveen *et al*., 2020).

Meanwhile, peripheral artery disease is one of the most common complications in T2DM. Previous studies reported that diabetic patients had a two to four-fold higher risk for developing peripheral artery diseases (e.g., critical limb ischemia [CLI]) than those with no T2DM ( Liao *et al*., 2013; Luscher *et al*., 2003). Of note, 500-1000 new cases of CLI per million people per year were estimated in the Western Europe and the United States ( Davies, 2012; Spreen *et al*., 2016). In an acute phase, peripheral artery disease can rapidly progress into acute limb ischemia (ALI), and such progression is expected to happen within two weeks of significant reduction of limb perfusion. Thus, such a condition can increase morbidity and mortality. Additionally, uncontrolled blood glucose also facilitates the worsening of ALI, and could potentially end up with limb amputation ( Fukuda *et al*., 2015; Kaur *et al*., 2020; Spreen *et al*., 2016; Waspadji, 2014).

Altogether, the hypercoagulability caused by both COVID-19 and T2DM predisposes ALI and therefore increases mortality. Thus, such an emerging and challenging situation requires immediate and prompt treatments. However, research about this particular topic and fixed guidelines to manage such complex conditions are currently limited ( Galanis *et al*., 2020; Kaur *et al*., 2020). Moreover, the presence of diabetic nephropathy could add more challenges in diagnosing and treating those patients. Here, we present a complex case of a patient with COVID-19, ALI, T2DM and diabetic nephropathy that was hospitalized and treated in a tertiary referral hospital in Indonesia.

## Case report

A 59-year-old Asian housewife was referred to the emergency department (ED) of Dr. Soetomo General Hospital (SGH) with bluish coloration, numbness and progressive pain in her left leg. The patient had been hospitalized in another hospital for shortness of breath due to COVID-19 two days before the referral, but because of the limb coloration, she was referred to SGH. Similar pain was also felt in her right leg, which was reddish in color. The patient had complained about the pain for five years (initially sensed as frequent tingling), which worsened one week before her visit at the hospital. The pain was intermittent and was mostly felt while moving or walking, and worsened at night during sleep. Most of the time, the patient hanged her legs down to alleviate the pain. During initial assessment, fever, occasional coughing and intermittent dyspnea were observed. The patient also complained about pain during micturition five days before the hospitalization. There were no issues regarding appetite and defecation. The patient also had a four-year history of uncontrolled T2DM with a bad compliance to oral antidiabetic (glimepiride 2 mg 1 – 0 – 0) and untreated hypertension. No relevant family medical history was reported.

**Figure 1.**
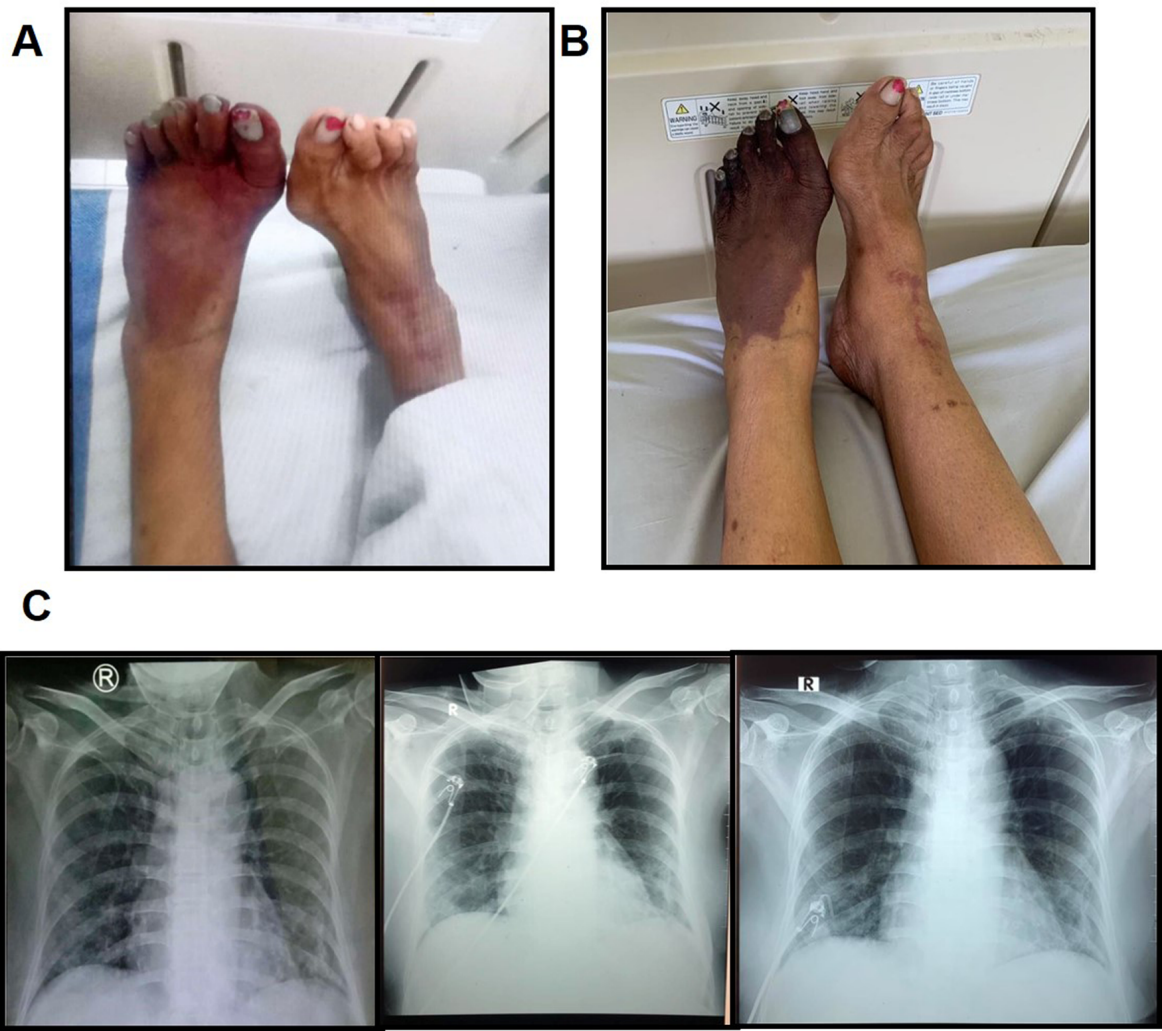
The progression of ALI and COVID-19-associated pneumonia. A) The clinical presentation of the lower extremities at day 0 of hospitalization. B) The worsening of ALI at day 8. C) The progress of COVID-19-associated pneumonia before referral, at day 1 and day 4 of hospitalization. Left: bilateral infiltrates were observed; middle: bilateral pneumonia was slightly increased; right: an improvement was visible.

The physical examinations at the ED showed a lethargic condition with Glasgow Coma Scale (GCS) of E4M5V6. Her blood pressure was 150/90 mmHg with heart rate of 110 beats/minute (bpm). Her respiratory rate was 24 times/minute, and her temperature was 36.7°C with blood oxygen saturation (SpO _2_) of 97%. Her pain was assessed using the Visual Analogue Scale (VAS) and resulted in a score of 6 out of 10. Her weight, height and body mass index (BMI) were 70 kg, 155 cm and 29.1, respectively. Head and neck examinations showed anemic conjunctivae with no icterus or cyanosis. Her cardiopulmonary examination was within a normal range (i.e., both sides of thorax were symmetrical and no retraction), as well as the abdominal examination (i.e., fluffy abdomen, bowel sounds were normal, liver and spleen were not palpable). Interestingly, her extremity assessment revealed a bluish coloration in her left leg with cold sensation during palpation and no pulse was detected ( Table 1). Detailed left leg examinations also discovered a necrotic region below her ankle, with a cold sensation up to one-third of her left lower extremity. Furthermore, the motoric and sensory functions of the left leg were poor. Meanwhile, her right leg was warm, had no necrosis, and exhibited normal sensory and motoric functions ( Figure 1A). Ankle Brachial Index (ABI) was negative on the left and 0.9 on the right.

**Table 1.** The pulsation status of the patient.

Artery	Right	Left
**Day 0**		
Femoral artery	++	++
Popliteal artery	++	++
Posterior tibial artery	+	-
Dorsalis pedis artery	+	-
**Day 7**		
Femoral artery	++	++
Popliteal artery	++	++
Posterior tibial artery	-	-
Dorsalis pedis artery	-	-
**Day 14**		
Femoral artery	++	++
Popliteal artery	++	Amputated
Posterior tibial artery	+	Amputated
Dorsalis pedis artery	-	Amputated

The laboratory examinations showed hemoglobin (Hb) of 10.8 g/dl, hematocrit of 35%, leucocyte count of 24460 /μl and platelet count of 627000/μl. The differential blood count of leucocytes highlighted neutrophil domination (88.1%) followed by lymphocytes (5%). Hemostatic parameters, such as prothrombin time (PT), activated partial thromboplastin time (aPTT), and D-dimer were 14.6, 30.8 and 9740 ng/ml, respectively. Her liver functions of aspartate aminotransferase (AST) and alanine aminotransferase (ALT) were 70 U/l and 193 U/l respectively. Blood albumin was 2.89 g/dl, fasting blood glucose was 128 mg/dl, HbA1c was 9%. Renal functions test revealed a serum creatinine of 1.5 mg/dl and blood urea nitrogen (BUN) of 61 mg/dl. Her C-reactive protein (CRP) was 22.5 mg/l, sodium 132 mg/dl, potassium 4.7 mg/dl, chloride 96 mg/dl. HbsAg, anti-HCV and HIV were all negative. COVID-19 polymerase chain reaction (PCR) test was positive. The urinalysis showed following results: nitrite positive, leucocytes positive, glucose 4+, A/C< 30, P/C<0,15, while her arterial blood gas analysis (BGA) showed pH 7.42, pCO2 38, pO2 146, HCO3 23.4 and SpO _2_ 99%. Additionally, her chest X-ray showed bilateral pulmonary infiltrates ( Figure 1C).

Taking into account the patient’s complaints, history, physical, laboratory and radiological examinations, the patient was then diagnosed with COVID-19-associated pneumonia with left inferior extremity dead limb and bilateral inferior extremity ALI, as well as urinary tract infection, anemia, hypoalbuminemia, T2DM and chronic kidney disease (CKD) stage III. Subsequently, she was treated with oxygen via simple mask at 6 liters per minute (lpm), sodium chloride (NaCl) 0.9% infusion 1000 ml/24 hours, subcutaneous heparin injection (2×5000 unit), intravenous (iv) dexamethasone injection (6 mg 1×1), remdesivir 1×200 mg, ceftriaxone 2×1 g, metronidazole 3×500 mg, insulin aspart (novorapid) 14 – 16 – 16, insulin detemir (levemir) 0 – 0 – 10, albumin 20% transfusion 100 ml/4 hours, oral isoprinosine 3×500 mg, oral vitamin D 1×5000 IU and oral cilostazol 1×100 mg. Her diet was also maintained within 1900 kcal/day.

At day four, the dyspnea was improved but the leg started to feel burnt and the pain progressed. Blood pressure was 149/84 mmHg, heart rate 68 bpm, respiratory rate 20 times/minute, SpO _2_ 98%, VAS score 5, COVID-19 PCR negative, leucocyte count was 27540 /μl with the proportion of neutrophil 87.4%, serum creatinine 1.4 mg/dl, blood albumin 2.8 mg/dl, D-dimer 6540 ng/ml, ferritin 1602 ng/ml, fibrinogen 642.6 mg/dl and procalcitonin (PCT) of 1.08. At day 5, the patient only complained about her progressive leg pain, while her daily examinations revealed blood pressure of 127/74 mmHg, heart rate 86 bpm, respiratory rate 20 times/minute, SpO _2_ 99% and VAS score of 5. Meanwhile, the COVID-19 PCR was negative, leucocyte count was 31390/μl with neutrophil 86.6%, serum creatinine 1.3 mg/dl, blood albumin 3 mg/dl, D-dimer 5570 ng/ml, ferritin 1572 ng/ml, fibrinogen 542 mg/dl and PCT of 0.23. Additionally, an improvement of the bilateral pulmonary infiltrates on chest X-ray was also observed ( Figure 1C).

**Figure 2.**
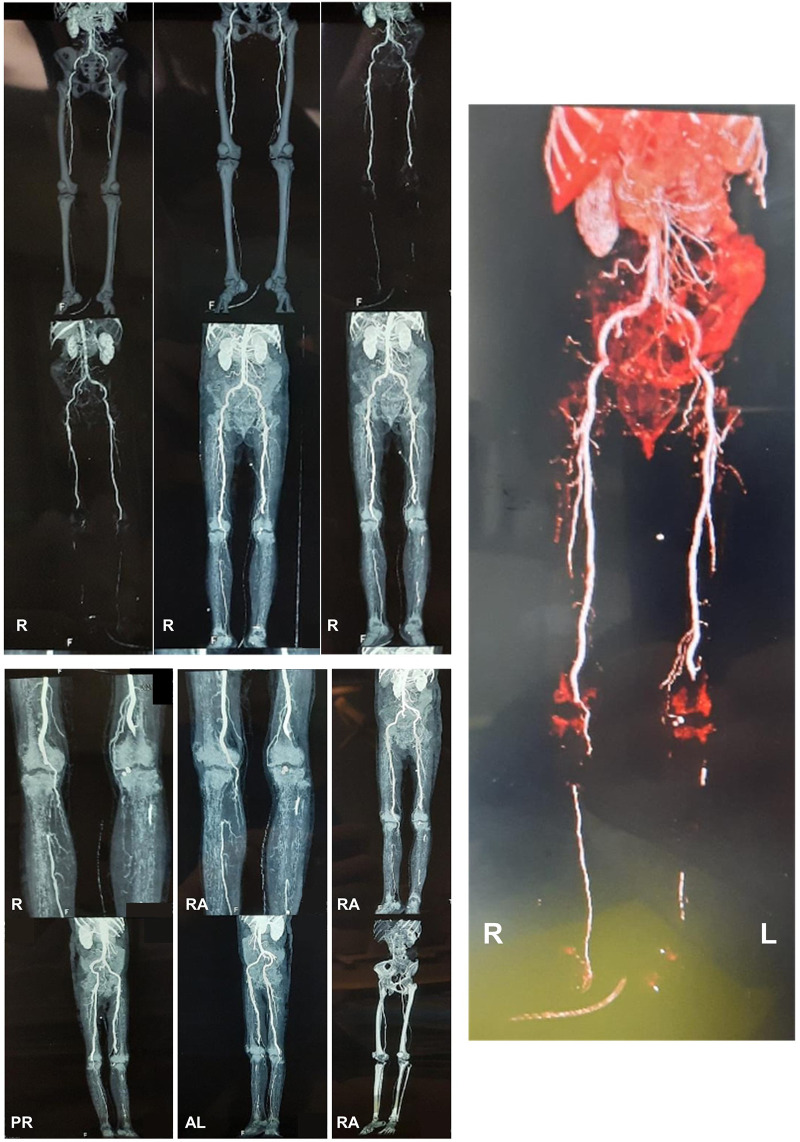
The computed tomography angiography (CTA) of the lower extremity. The CTA revealed an abdominal aortosclerosis with abnormalities on both limbs. Right limb: total occlusion due to 6 cm thrombus on right popliteal artery (from 1 cm above right femorotibial joint toward inferior), right anterior tibial artery received contrast flow from collateral arteries and no contrast flow was seen in right posterior tibial, peroneal and dorsalis pedis arteries. Left limb: total occlusion due to 12.7 cm thrombus on left popliteal artery (from 6 cm above left femorotibial joint toward inferior). No contrast flow was seen in left anterior tibial, posterior tibial, peroneal and dorsalis pedis arteries. (This figure has been edited in Microsoft PowerPoint 2016 to obscure patient’s data.)

At day 7, the patient complained that the pain in her leg had increased significantly. Her blood pressure was 130/80 mmHg, heart rate 98 bpm, respiratory rate 20 times/minute and SpO _2_ 98%. The urine culture was positive for *Escherichia coli* sensitive to cefoperazone sulbactam. Because of the progressive extremity pain, the patient was referred to the cardiothoracic and vascular surgery (CTS) division and, consistent with the first diagnosis, she was diagnosed with left dead limb and bilateral ALI ( Figure 1B), and was advised to perform computed tomography angiography (CTA) and undergo an above-knee amputation. At day 9, the right lower extremity CTA ( Figure 2) discovered total occlusion due to 6 cm thrombus on right popliteal artery (from 1 cm above right femorotibial joint toward inferior). Right anterior tibial artery received contrast flow from collateral arteries and no contrast flow was seen in right posterior tibial, peroneal and dorsalis pedis arteries. Meanwhile, the left lower extremity CTA showed total occlusion due to 12.7 cm thrombus on left popliteal artery (from 6 cm above left femorotibial joint toward inferior). Moreover, no contrast flow was seen in left anterior tibial, posterior tibial, peroneal and dorsalis pedis arteries.

**Figure 3.**
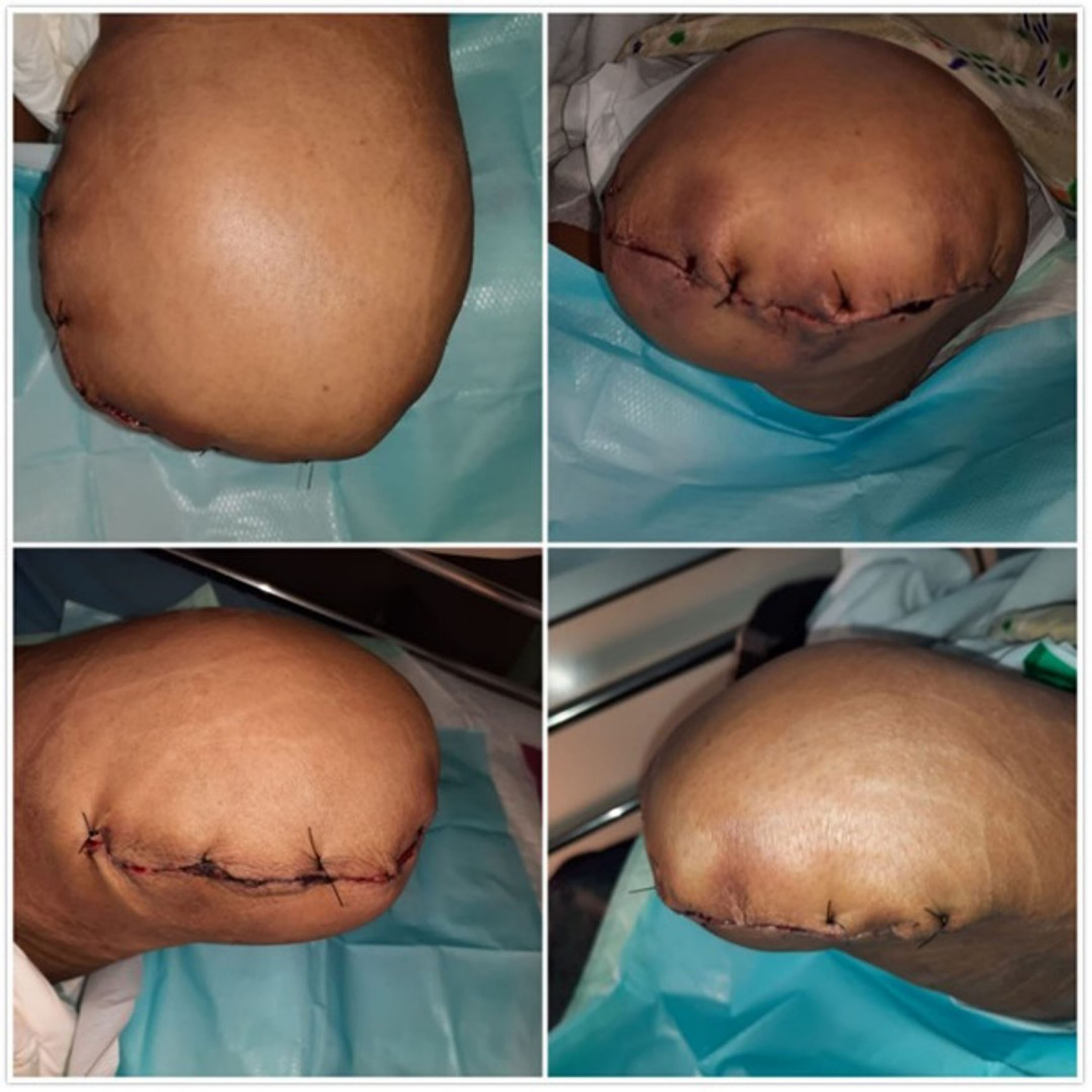
The appearance of left lower limb post amputation. Two days post-surgery, the patient was safely discharged and followed up regularly for recurring symptoms at the outpatient clinic.

At day 11, the patient was still waiting for surgery and the pain increased. Her blood pressure was 130/80 mmHg, heart rate 88 bpm, respiratory rate 20 times/minute, SpO _2_ 98% and VAS equals 7. Laboratory parameters showed Hb 10.3, leucocyte count 22610/μl, platelet 509000/μL, D-dimer 3960 ng/ml and random blood glucose of 171. Subsequently, the patient received 8 lpm oxygen through a simple mask, diet type-B 1900 kcal/24 hours, iv fluid with NaCl 0.9% 1000 ml/24 hours, cefoperazone sulbactam 2×1 g iv, subcutaneous novorapid 14 – 16 – 16 unit, subcutaneous levemir 0 – 0 – 10 unit, subcutaneous heparin 2×5000 unit and oral cilostazol 1×100 mg. At day 13, her blood pressure was 127/70 mmHg, heart rate 90 bpm, respiratory rate 20 times/minute and SpO _2_ 98%. Laboratory results showed Hb 9, leucocyte count 11800/μL, platelet 410000/μL, BUN 20 mg/dl, serum creatinine 1.3 mg/dl and random blood glucose of 160. At this point, the patient was assigned for antegrade right femoral artery thrombectomy and left above-knee amputation at the following day. At day 14, the surgeries (both amputation and thrombectomy) were performed ( Figure 3) and 10 cm thrombus was retrieved in the right femoral artery. Lastly, as a follow-up, at day 17, no symptoms were observed, therefore the patient was discharged.

## Discussion

ALI is a pathological condition in which the perfusion toward extremities suddenly and significantly drops. Such condition often threatens the viability of the extremity, with a high risk of limb loss if the perfusion is completely obstructed. In most cases, the disease rapidly progresses within two weeks of the initial complaint. Therefore, optimal history-taking and physical examinations are crucial in ALI diagnosis and treatment. Cold sensation in extremity (poikilothermy), pain, pallor, pulselessness, sensory disturbance (paresthesia) and motoric alteration (paralysis) are commonly found in the disease trajectory of ALI ( Gerhard-Herman *et al*., 2017; Obara, Matsubara, & Kitagawa, 2018). Additionally, limb examinations, including the pulsation palpation and ABI assessment, are essential. Due to the complex pathophysiology of the disease and the interplay with other comorbidities, the risk factors of ALI (e.g., T2DM, hypertension, smoking, chronic pulmonary diseases and CKD) also need to be explored ( Fukuda *et al*., 2015).

The American College of Cardiology (ACC) and American Heart Association (AHA) proposed that ALI can be divided into 3 categories: first grade, viable body parts with no immediate threat; second grade, threatened body parts (IIa: marginally threatened, indicating that body parts can be salvaged if immediate and prompt treatments were performed; IIb: immediately threatened, indicating that threatened body parts require immediate revascularization); and third grade, irreversible damage of body parts ( Gerhard-Herman *et al*., 2017). In the case we presented above, we can classify the left lower extremity of the patient as third grade because no pulsation was observed in left posterior tibial and dorsalis pedis arteries. Moreover, the SpO _2_ was negative on the left lower limb with some necrotic regions, indicating the nonviability of the left lower limb. Meanwhile, the right lower extremity can be classified in the second grade because the pulsations and SpO _2_ persisted. Adjunctive (radiological) examinations, such as duplex ultrasound, CTA or magnetic resonance angiography (MRA) could also assist the diagnosis of ALI by locating the obstruction and guide revascularization procedure ( Davies, 2012; Kinlay, 2016). Studies have shown that CTA is 89-99% sensitive and 83-97% specific for ALI ( Fukuda *et al*., 2015).

In this case, we also saw that the rapid progression of ALI happened after the SARS-CoV-2 infection. COVID-19 often induces a hypercoagulable state and hyperinflammation which could progress into disseminated intravascular coagulation (DIC) ( Asakura & Ogawa, 2021). Such a condition is commonly marked by the elevation of D-dimer and fibrinogen ( Eljilany & Elzouki, 2020). On the other hand, SARS-CoV-2 infection can also damage the endothelial cells, causing endothelial dysfunction and initiate the expression of tissue factors, activate platelets, and increase von Willebrand factor and factor VIII ( Bonaventura *et al*., 2021; Evans *et al*., 2020). Altogether, they facilitate the formation of thrombin and blood clot (thrombus). Thrombin triggers inflammation through its platelet modulation and stimulation of neutrophil, which further activates endothelial cells and monocytes, allowing the formation of micro thrombus. Such condition can increase the thromboembolic risk in designated locations.

Interestingly, COVID-19 is able to instigate hyperglycemia, even without previous history of T2DM. Such hyperglycemic condition causes oxidative stress, which increases inflammation and coagulation. This increases the risk of coagulation and facilitates thrombosis as well ( Ceriello, 2020). Due to the complex interactions, the prognosis of peripheral artery diseases in T2DM relies on the comorbidities, the presence of concomitant infections, neuropathy and the patient’s immune status. Uncontrolled T2DM could speed up the atherosclerosis and was shown to have a five to 10-fold higher risk of amputation ( Li *et al*., 2007; Serrano Hernando & Martin Conejero, 2007).

In general, the management of first grade ALI comprises pharmacological treatments, while the second grade requires revascularization and presumably amputation if the limb cannot be salvaged. Common drugs for peripheral artery diseases, such as antiplatelets, statins, antihypertensives, anticoagulants, cilostazol and pentoxifylline could be administered in ALI, together with lifestyle modifications to control blood glucose level and smoking cessation.

The presented case highlighted the effectiveness of revascularization and limb amputation in treating end-stage ALI (grade IIb and III) in patients with complex diseases such as T2DM, diabetic nephropathy and COVID-19. This case also exemplified that anticoagulation might not be adequate to treat ALI in the presence of COVID-19 and T2DM, and that early revascularization should be considered in such situations. Nonetheless, the COVID-19 infectivity has to be taken into account to maintain the safety of the healthcare workers during the procedure. In the presented case, we had to wait until we obtained two-times negative COVID-19 PCR results before sending the patient for CTA and surgical interventions. This local policy could be called a ‘double-edged sword’, in that on the one hand it protected the healthcare workers from COVID-19, but on the other hand, it delayed the procedure for the patient and facilitated the progression of ALI. In the future, an improved understanding of COVID-19 infectivity and pathogenesis would hopefully reduce the time to interventions and diminish ALI-associated morbidity and mortality.

## Conclusion

Here, we presented a 59-year-old female with COVID-19, T2DM, third grade ALI and CKD stage III (diabetic nephropathy). In this patient, revascularization of the right lower extremity via thrombectomy and left lower limb amputation were performed based on findings in physical examinations, supported by CTA. At day 17, the patient was discharged and recovered from COVID-19, confirmed by two negative PCR tests. This case highlights the complexity of ALI in the presence of COVID-19 and comorbidities (i.e., T2DM and diabetic nephropathy), which requires intensive monitoring and prompt immediate treatments to reduce morbidity and mortality.

## Consent

A written informed consent for the publication of her clinical details and clinical images was obtained from the patient.

## Data availability

All data underlying the results are available as part of the article and no additional source data are required.
