# Supplementary material for: Case Report: Diabetic nephropathy aggravates the progression and prognosis of COVID-19-associated acute limb ischemia
Source: F1000Res. 2021 Sep 17;10:584. Originally published 2021 Jul 16. [Version 2] doi: 10.12688/f1000research.54193.2 (PMC8430448; doi:10.12688/f1000research.54193.2)
Supplement: Peer review history

# Peer review: Reviewer response for version 2

**DOI:** 10.5256/f1000research.77605.r94693

**Published:** 2021-09-20

**Authors:** Ketut Suastika

**Affiliations:** 1 Faculty of Medicine, Sanglah General Hospital, Udayana University, Bali, Indonesia

I have no more comments, the suggestion has been revised by the authors. Thank you.

Are enough details provided of any physical examination and diagnostic tests, treatment given and outcomes?

Yes

Is the case presented with sufficient detail to be useful for other practitioners?

Yes

Is sufficient discussion included of the importance of the findings and their relevance to future understanding of disease processes, diagnosis or treatment?

Yes

Is the background of the case’s history and progression described in sufficient detail?

Yes

Reviewer Expertise:

Clinical and adult endocrinologist

I confirm that I have read this submission and believe that I have an appropriate level of expertise to confirm that it is of an acceptable scientific standard.

# Peer review: Reviewer response for version 1

**DOI:** 10.5256/f1000research.57653.r92346

**Published:** 2021-09-01

**Authors:** Joyce Bernardi

**Affiliations:** 1 Biomedical Research and Translational Medicine, Masonic Medical Research Institute, Utica, NY, USA

The authors describe in detail the case of a 59-year-old female with T2DM, presenting with bilateral acute limb ischemia that aggravated after COVID-19 infection. A revascularization of the right lower limb through thrombectomy and amputation of the necrotic left lower limb have been performed. The manuscript highlights how the presence of COVID-19 infection and other comorbidities is critical in the evolution of ALI and how a timely intervention is important to reduce morbidity and mortality.

 The background of the case is well described, together with an extensively detailed description of the progression, diagnosis, and treatment. In the discussion the authors give a good overview of what is known about the interaction between COVID-19 and other comorbidities, such as T2DM and ALI, pointing out the importance of a prompt intervention.

Are enough details provided of any physical examination and diagnostic tests, treatment given and outcomes?

Yes

Is the case presented with sufficient detail to be useful for other practitioners?

Yes

Is sufficient discussion included of the importance of the findings and their relevance to future understanding of disease processes, diagnosis or treatment?

Yes

Is the background of the case’s history and progression described in sufficient detail?

Yes

Reviewer Expertise:

cardiac electrophysiology

I confirm that I have read this submission and believe that I have an appropriate level of expertise to confirm that it is of an acceptable scientific standard.

# Response

**Published:** 2021-09-10

**Authors:** Henry Sutanto

**Affiliations:** CARIM School for Cardiovascular Diseases, Maastricht University, The Netherlands

*The authors describe in detail the case of a 59-year-old female with T2DM, presenting with bilateral acute limb ischemia that aggravated after COVID-19 infection. A revascularization of the right lower limb through thrombectomy and amputation of the necrotic left lower limb have been performed. The manuscript highlights how the presence of COVID-19 infection and other comorbidities is critical in the evolution of ALI and how a timely intervention is important to reduce morbidity and mortality. The background of the case is well described, together with an extensively detailed description of the progression, diagnosis, and treatment. In the discussion the authors give a good overview of what is known about the interaction between COVID-19 and other comorbidities, such as T2DM and ALI, pointing out the importance of a prompt intervention.*

**Response: **We would like to thank the reviewer for the time and willingness to assess the quality of our manuscript.

# Peer review: Reviewer response for version 1

**DOI:** 10.5256/f1000research.57653.r91878

**Published:** 2021-08-16

**Authors:** Ketut Suastika

**Affiliations:** 1 Faculty of Medicine, Sanglah General Hospital, Udayana University, Bali, Indonesia

The Case Report was reported detailly on clinical findings, supporting diagnosis examinations, and management of the patients. It was also written well. The case report has become a reminder that in the patient with T2DM and CPVID-19, morbidity or mortality does not come from lung complications only. 

 To the authors: please note the value of eGFR by formula calculation to know the degree of CKD stage.

Are enough details provided of any physical examination and diagnostic tests, treatment given and outcomes?

Yes

Is the case presented with sufficient detail to be useful for other practitioners?

Yes

Is sufficient discussion included of the importance of the findings and their relevance to future understanding of disease processes, diagnosis or treatment?

Yes

Is the background of the case’s history and progression described in sufficient detail?

Yes

Reviewer Expertise:

Clinical and adult endocrinologist

I confirm that I have read this submission and believe that I have an appropriate level of expertise to confirm that it is of an acceptable scientific standard.

# Response

**Published:** 2021-09-10

**Authors:** Henry Sutanto

**Affiliations:** CARIM School for Cardiovascular Diseases, Maastricht University, The Netherlands

- *The Case Report was reported detailly on clinical findings, supporting diagnosis examinations, and management of the patients. It was also written well. The case report has become a reminder that in the patient with T2DM and CPVID-19, morbidity or mortality does not come from lung complications only. *
- **Report: **We would like to thank the reviewer for the time and willingness to assess the quality of our manuscript.
- *To the authors: please note the value of eGFR by formula calculation to know the degree of CKD stage.*
- **Report: **In the new version of the manuscript, we have added the estimated Glomerular Filtration Rate (eGFR) during admission (38 ml/min/1.73 m ^2 ^or stage 3B) and before discharge (45 ml/min/1.73 m ^2^ or stage 3A), which indicated an improvement of the diabetic nephropathy after treatments.
